# Effects of Customized Full-Contact Insoles Crafted with Polyester Fabric Sheets on Plantar Pressure and Gait in Hallux Valgus

**DOI:** 10.3390/bioengineering12020103

**Published:** 2025-01-23

**Authors:** Hsin-Yu Chen, Chin-Kang Chang, Fu-Ting Wang, Chia-Hao Yen, Hsiang-Chun Chuang, Tsung-Yang Wang, Fang-Yao Chiu, Hsien-Te Peng

**Affiliations:** 1 Rehabilitation and Technical Aid Center, Taipei Veterans General Hospital, Taipei 112, Taiwan; sychen2@vghtpe.gov.tw (H.-Y.C.); cjk2944@yahoo.com.tw (C.-K.C.); ftwang@vghtpe.gov.tw (F.-T.W.); chyen3@vghtpe.gov.tw (C.-H.Y.); hcchuang3@vghtpe.gov.tw (H.-C.C.); tywang8@vghtpe.gov.tw (T.-Y.W.); 2 Department of Orthopedics, Taipei Veterans General Hospital, Taipei 112, Taiwan; fychiu@vghtpe.gov.tw; 3 Department of Physical Education, Chinese Culture University, Taipei 111, Taiwan

**Keywords:** hallux valgus, customized foot orthotic, insole, plantar pressure

## Abstract

This study investigates the benefits of innovative full-contact insoles, crafted using polyester fabric sheets of thermoplastic extruded materials, for individuals with hallux valgus-induced plantar pain. Thirty-five individuals with hallux valgus-induced foot pain were randomly allocated to either the experimental group, wearing innovative full-contact insoles 6 h daily, or the control group, using regular foot insoles, over a period of 12 weeks. Data collection occurred at baseline, and at 1 week, 2 months, and 3 months after the initial fitting. Results indicated that the innovative full-contact insoles significantly reduced anteroposterior displacement by an average of 0.9 cm (*p* = 0.025) and displacement area by 0.79 cm^2^ (*p* = 0.012). Gait improvements included an increase in the swing phase (36.46%, *p* = 0.008) and a reduction in stance phase duration (63.54%, *p* = 0.019). Pressure peaks at critical foot regions like the second metatarsal and medial heel were reduced by up to 39.45 kPa (*p* = 0.016) and 104.07 kPa (*p* = 0.031), while contact areas in the midfoot increased by 6.17 cm^2^ (*p* = 0.039). Foot pain decreased by an average score of 1.28 points on a 10-point scale across various measures (*p* = 0.041). These findings suggest that the innovative full-contact insoles effectively enhance pressure distribution and gait dynamics in patients with hallux valgus, providing a viable option for managing foot discomfort.

## 1. Introduction

Patients with hallux valgus present complex foot structure alterations, including forefoot widening, lateral deviation of the first metatarsal, medial deviation of the hallux, and forefoot abduction, assessed by the hallux valgus angle [1]. Compared to those with normal feet, individuals with hallux valgus often experience decreased hallux peak pressure, contact time, contact area, and time-integrated pressure during walking, indicating compromised hallux function [2]. Elevated peak pressure in the second metatarsal is common, leading to increased forefoot loading and metatarsal pain [2,3].

Customized full-contact insoles redistribute pressure, relieving foot pain by shifting peak pressure from the metatarsal and heel to the proximal metatarsal and lateral midfoot [4]. They increase the contact area and provide arch support, significantly reducing forefoot plantar pressure and ensuring even pressure distribution [5,6]. In the previous studies, the full-contact insoles described are typically custom-made through a molding process using an impression of the wearer’s foot [7,8,9,10]. Medial-fixed, full-contact insoles mitigate rearfoot pronation, reducing peak pressure beneath the toes, lateral metatarsals, lateral foot, and heel [7]. They enhance midfoot pressure and first metatarsophalangeal joint force, improving user comfort [8,9]. Appropriate insole design is essential due to interpopulation force differences and varied foot pathologies; customized insoles outperform generic flat insoles in comfort and effectiveness [10].

Some studies have utilized insoles that are created using 3D scanning to build a model of the foot [7]. Others have collected barefoot plantar pressure data using pressure sensors, and then produced full-contact insoles using 3D printing based on the pressure distribution [9]. A more traditional method involves creating foot models from phenolic foam boxes for casting [11].

Commercially available full-contact insoles often lack customization, relying on standardized forefoot shapes that may not adequately accommodate individual foot characteristics [12]. For users with specific conditions, such as hallux valgus or other unique foot deformities, commercially available corrective insoles may not be suitable. However, using fully customized insoles comes with its own challenges, including high costs and time-consuming production processes. Typically, some insoles utilize foam fillings to adjust thickness and shape [5,13]. Moreover, the foam fillings tend to deform with prolonged use, necessitating the purchase of new insoles as replacements [14].

In this study, an innovative approach was introduced by utilizing polyester fabric sheets composed of thermoplastic extruded materials for the fabrication of insoles and structural support, representing a departure from traditional filling methods. We developed a customized insole using an alternative method, anticipated to deliver comparable benefits to those demonstrated in the aforementioned studies, particularly in managing pain, a major clinical implication for patients with hallux valgus. This study specifically aimed to evaluate the effects of customized innovative full-contact insoles (IFCIs) on reducing pain and enhancing the center of pressure deviation during static standing, in addition to improving plantar pressure distribution and gait patterns in patients with hallux valgus. The IFCIs, manufactured using advanced fabrication processes, were compared with flat insoles (FIs). It was hypothesized that users of IFCIs would exhibit altered plantar pressure, gait parameters, contact area, and significantly reduced pain after 1 week, 2 months, and 3 months, relative to baseline measurements (week 0).

## 2. Materials and Methods

### 2.1. Participants

This study recruited 35 individuals experiencing forefoot pain or plantar discomfort associated with hallux valgus. Inclusion criteria comprised individuals aged ≥ 20 years, presenting with metatarsal pain or moderate hallux valgus (20 to 40°), capable of independent walking, without visual or auditory impairment, and willing to adhere to wearing insoles as instructed. Exclusion criteria included any neurological disorder affecting gait, severe cardiac or pulmonary conditions hindering walking, diabetic peripheral neuropathy, history of fractures or joint replacements affecting lower limb length, osteoporosis or spinal compression fracture, foot, ankle, or knee joint deformities, soft tissue-related problems hindering testing, cognitive impairment, and history of psychiatric conditions or substance abuse. Participants were randomly assigned to either the experimental group (IFCI group [IFCIG]) or the control group (FI group [FIG]). The IFCIG wore IFCIs for 12 weeks (6 h daily), while the FIG wore FIs. Approval for the study was obtained from the Ethics Committee of Taipei Veterans General Hospital, and informed consent was obtained from all participants.

### 2.2. Manufacturing of Customized IFCIs

**Figure 1:**
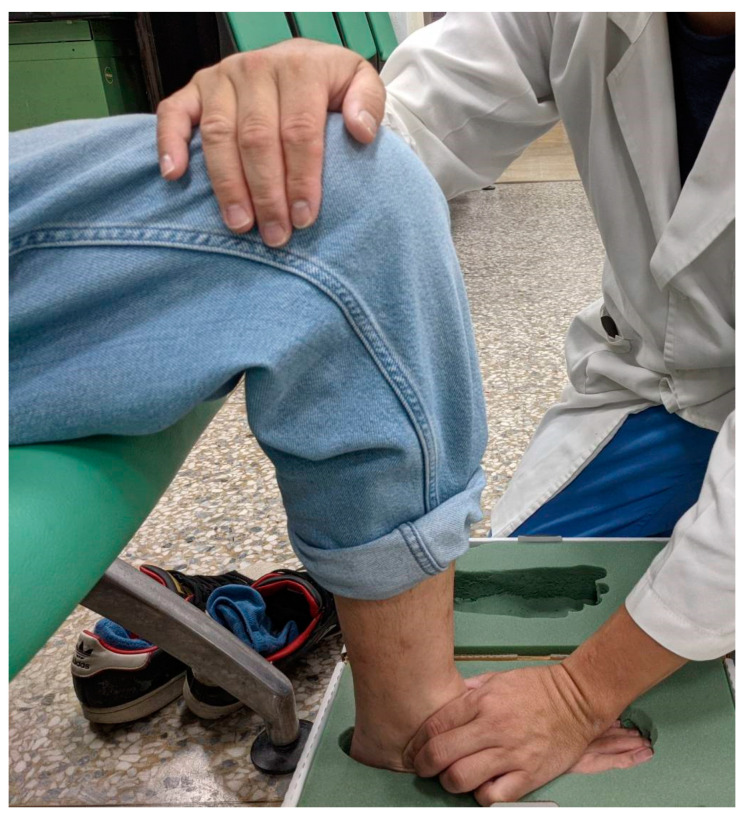
Pose while capturing foot impressions.

**Figure 2:**
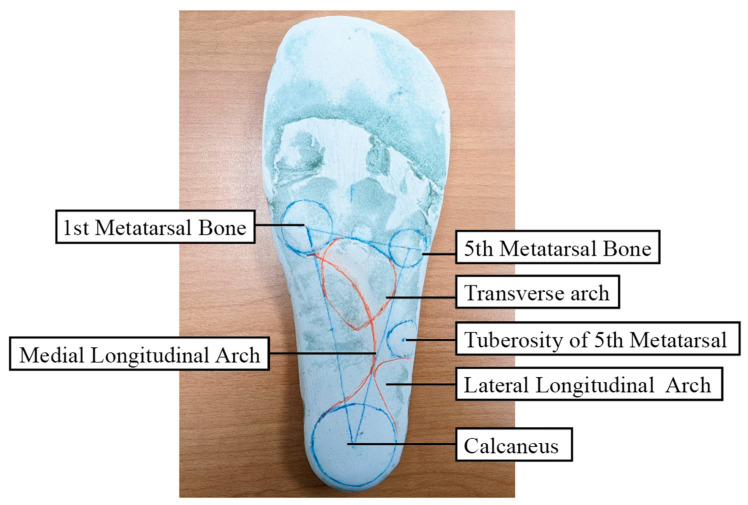
Positive cast with marks and lines.

In this study, IFCIs were meticulously crafted through an innovative process by skilled prosthetists and orthotists at the Rehabilitation Center for the Disabled of Taipei Veterans General Hospital. These insoles were custom-tailored to each participant’s unique foot anatomy. To capture foot impressions, participants were instructed to sit in a relaxed position with knees bent at a 90° angle and ankles centered. The selection of this semi-weight bearing position for casting the foot impressions is predicated on its appropriateness for capturing the structural characteristics of common foot types, particularly those with low or collapsed arches. This casting technique is advantageous as it records the arch’s configuration prior to any collapse that may occur under full weight bearing. Moreover, it allows for a moderate degree of pronation during the impression process, which is critical for accurately modeling the foot’s natural role in shock absorption. This method ensures that the custom insoles provide optimal support and functionality by maintaining the integrity of the foot’s arch and its biomechanical alignment during dynamic activities. Using a foam molding box, foot impressions were obtained and filled with plaster to create a positive cast (Figure 1). The positions of the metatarsal bone heads, heel center point, and the line connecting the second metatarsal bone were marked on the cast. Subsequently, the transverse, medial longitudinal, and lateral longitudinal arches were meticulously formed to maintain a normal arch position (Figure 2).

The insoles were crafted using specific materials and a precise manufacturing process. Initially, the positive cast was placed into a molding machine (Trautman Vacuum Station, SPS, Alpharetta, GA, USA), where three layers of materials were systematically applied onto the cast and molded under vacuum heating. The first layer consisted of a soft, perforated ethylene vinyl acetate material (2 mm thickness, Schein Orthopädie Service KG, Remscheid, Germany), heated to 100 °C for 20 s. The second layer comprised a polyester fabric sheet of thermoplastic extruded materials with thermoadhesive on both sides (Relion G18 Rigid, TECNOGI S.p.A., Borgolavezzaro, Italy) with a thickness of 1.55–1.65 mm and covering the full-foot size, heated to 100 °C for 3 min. The third layer was another polyester fabric sheet of thermoplastic extruded materials with thermoadhesive on both sides (Relion G12 Rigid, TECNOGI S.p.A., Borgolavezzaro, Italy), thinner at 1.1–1.2 mm thickness and covering two-thirds of the foot size, heated to 100 °C for 1 min. These materials, commonly used in shoe counters for structural support, become pliable and adhesive upon heating, allowing for angular adjustments during the vacuum molding process.

**Figure 3:**
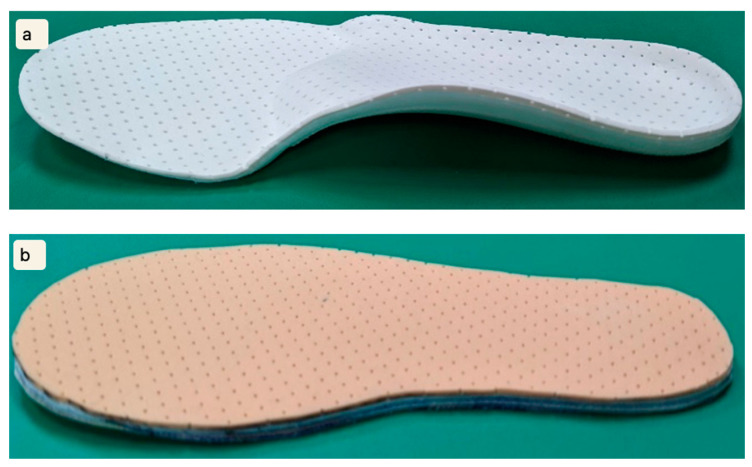
Insoles: (**a**) innovative full-contact insole; (**b**) flat insole.

IFCIs (Figure 3) are tailored for individuals with hallux valgus, offering customized arch support to address associated abnormalities. Unlike standard commercial insoles using standardized forefoot shapes and foam materials for correction [5,12,13], our insoles utilize molded plastic sheets for rapid shaping, material efficiency, and precise support tailored to individual foot characteristics. This personalized approach caters specifically to individual needs, distinguishing our insoles from commercial alternatives. The manufacturing process is streamlined, involving trimming of the first layer of ethylene vinyl acetate material to shape the insole. They are thinner and lighter than traditional custom-made insoles, suitable for various shoe styles. They provide robust support for both the medial and lateral arches, with enhanced support in the transverse arch for improved overall foot support [15]. The flexible fiberboard material ensures a snug fit to the arch shape, providing support and shock absorption during walking [16]. Moreover, the sandwich structure allows for easy modifications during manufacturing.

### 2.3. FIs

FIs were prepared using the same materials and processes as those used for the customized IFCIs (Figure 3), with the exception of customization. In this case, the insole from each participant’s shoes served directly as a size reference.

### 2.4. Experimental Procedure

During the initial test (pretest; week 0), all the participants wore standard sports shoes (model: ARWR92280; ARNOR, Taipei, Taiwan) equipped with FIs made using the same material as that used for the customized IFCIs. The participants were instructed to walk comfortably and steadily along a 5 m test walkway, starting from a designated point. Data were collected from five trials; for each trial, we excluded the first two steps and then analyzed the remaining six steps taken during the gait test. Thus, a total of 30 steps were analyzed [17,18]. After the test, the participants were evaluated for pain by using the Taiwanese version of the Brief Pain Inventory (BPI) [19].

**Figure 4:**
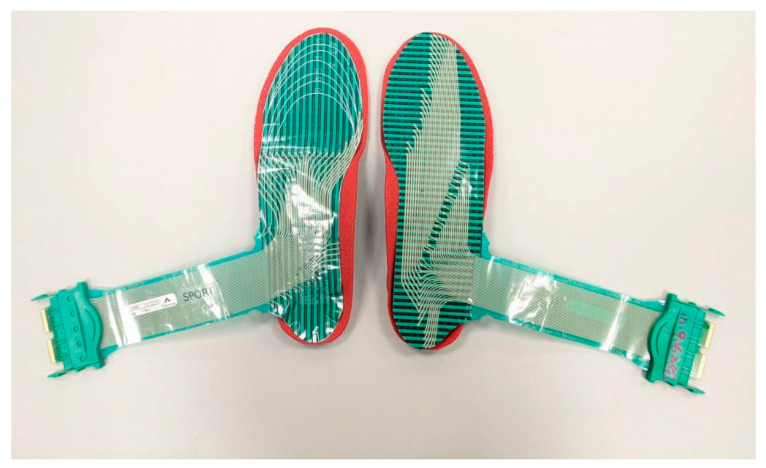
Foot pressure system and insoles.

**Figure 5:**
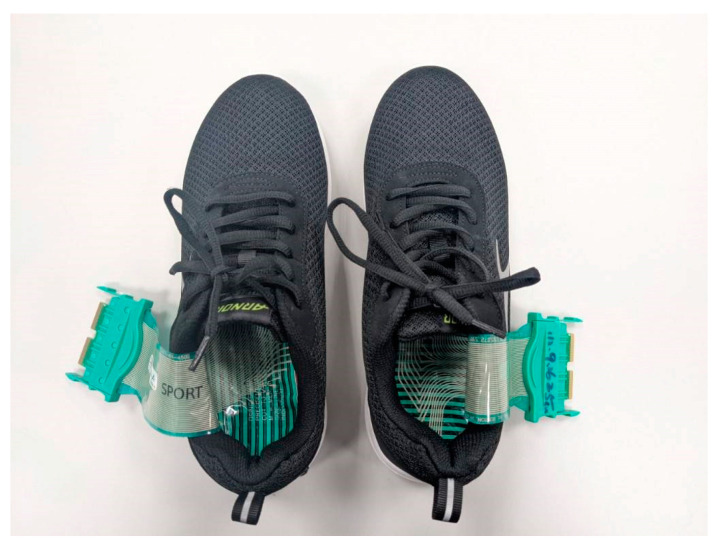
Foot pressure system and insoles in the shoes.

**Figure 6:**
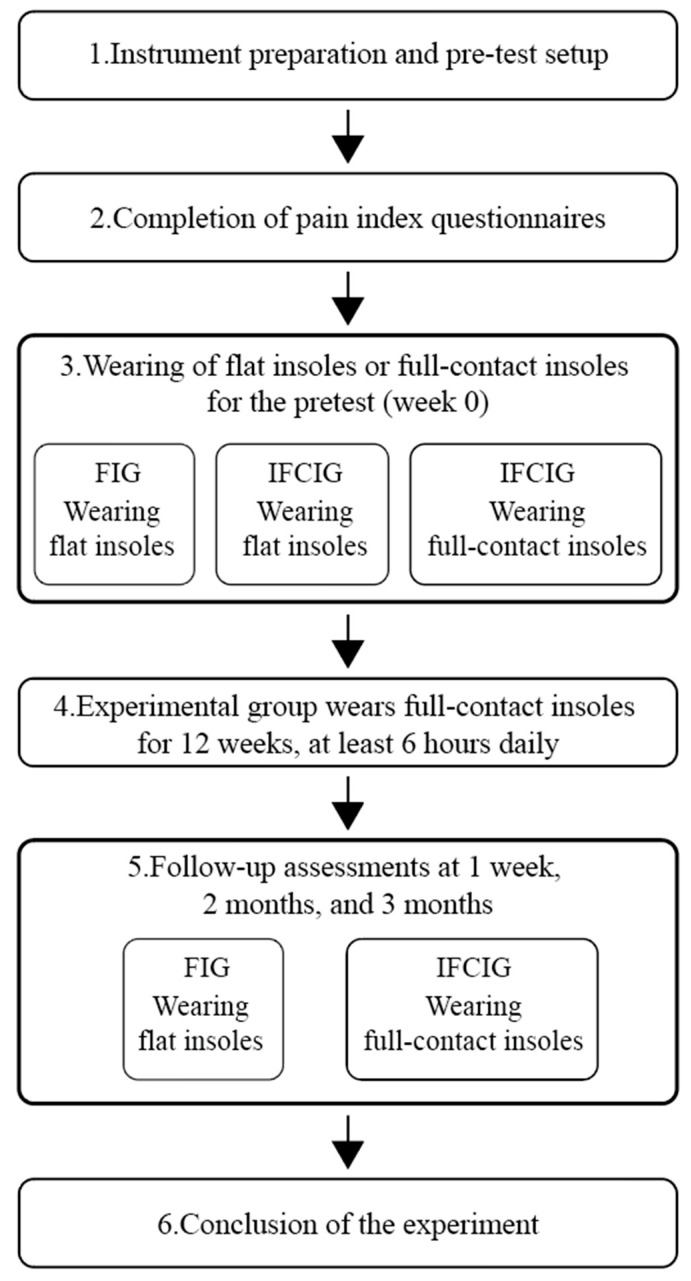
Experimental flowchart.

After the FI test, the participants in the IFCIG switched to shoes equipped with IFCIs; the test was conducted using the same protocol. With the same protocol, follow-up assessments were conducted 1 week, 2 months, and 3 months after the initial test (Figure 4). All participants wore the same footwear for at least 12 weeks (during the study) (Figure 5); the insoles were used for at least 6 h per day. The experimental flowchart is shown in Figure 6.

### 2.5. Data Collection

Data on gait parameters related to foot pressure distribution were collected using a wireless plantar pressure sensing system (Tekscan, Boston, MA, USA) [20], with a sampling frequency of 60 Hz. Simultaneously, data were collected using the GAITRite Gait Analysis System (GAITRite Gold; CIR Systems, NJ, PA, USA) [21], which includes an electronic walkway mat (length: 4.6 m; width: 0.9 m; effective area: 3.6 m × 0.6 m; sensor count: 13,824). Calibration of the equipment was performed using the participants’ body mass, requiring each participant to stand on each foot for seven seconds on both the Tekscan pressure sensor and the GAITRite walkway, ensuring the accuracy and reliability of the measurements.

To assess balance, participants stood on a footplate (Currex, Hamburg, Germany) with both feet while focusing on a target object located 3 m away for 30 s, with their arms hanging naturally by their sides. This procedure was repeated three times. Data were sampled at a frequency of 100 Hz [22]. The footplate was calibrated by participants’ body mass.

### 2.6. Data Analysis

**Figure 7:**
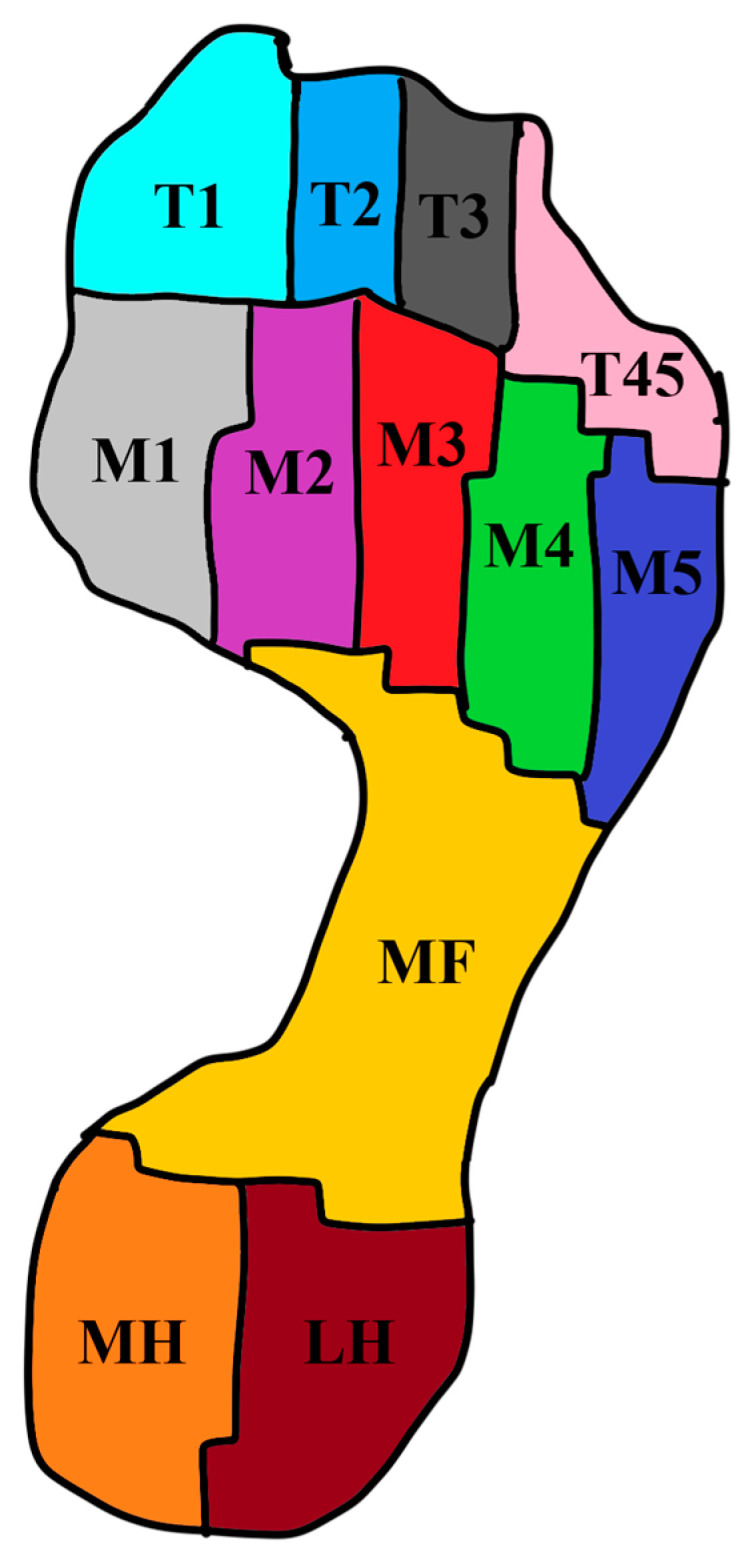
Pressure regions on the foot (T1, hallux; T2, second toe; T3, third toe; T45, fourth and fifth toes; M1, first metatarsal; M2, second metatarsal; M3, third metatarsal; M4, fourth metatarsal; M5, fifth metatarsal; MF, midfoot; MH, medial heel; LH, lateral heel).

The foot-pressure parameters analyzed included peak pressure and contact area across specific foot regions, such as the toe (hallux, toes 2 to 5), forefoot (first metatarsal, metatarsals 2 to 5), midfoot, and rearfoot (lateral and medial heels) (Figure 7) [9]. The following gait parameters were analyzed: walking velocity, cadence and swing, stance, single support, double support phases during stride cycles. Additionally, parameters related to the center of pressure during 30 s standing were obtained, including anteroposterior and mediolateral displacement of the center of pressure for both the left and right feet, as well as the area of deviation calculated from the anteroposterior and mediolateral deviations (see Equation (1)). In participants experiencing pain in both feet, the foot with more pain or the dominant foot was selected for pain assessment.

Equation (1): (where π is the mathematical constant pi; a and b are the anteroposterior and mediolateral displacement values, respectively.)

π × a × b(1)

### 2.7. Statistical Analysis

Participant demographics and other parameters were summarized using descriptive statistics (mean (standard deviation)). Statistical analyses were performed using SPSS (version 18.0; SPSS, Chicago, IL, USA). Data normality was assessed using the Shapiro–Wilk test, which revealed non-normal distribution. Accordingly, nonparametric statistical methods were employed for analysis. The Mann–Whitney U test, a nonparametric statistical method, was employed to compare differences between the IFCIG and FIG groups. Intragroup changes at different time points were analyzed using the Friedman test. Post hoc comparisons were conducted using the Wilcoxon signed-rank test. The significance level was set at α = 0.05. Nonparametric effect sizes (ES; calculated using Equation (2)) were determined to assess the magnitude of differences for various parameters between FIs and IFCIs. ES values were interpreted as follows: r = 0.1 for small effects, r = 0.3 for medium effects, and r = 0.5 for large effects.

Equation (2):

r = z/√n(2)

## 3. Results

The IFCIG and FIG comprised 19 and 16 participants, respectively, with mean ages of 53.99 (12.73) and 54.69 (15.21) years, heights of 159.92 (8.17) and 162.56 (6.84) cm, body mass of 58.79 (10.46) and 58.84 (8.85) kg, and hallux valgus angles of 27.00° (10.87°) and 29.94° (9.48°). No initial differences were noted between groups, indicating homogeneity.

In terms of gait characteristics (Table 1), the IFCIG demonstrated significant increases in swing phase and decreases in stance phase at the 1-week follow-up (ES: 0.6 [*p* = 0.008] and 0.6 [*p* = 0.019], respectively). By the 2-month follow-up, the IFCIG displayed significant reductions in stance and double support phases, alongside a significant increase in swing phase (ES: 0.64 [*p* = 0.025], 0.67 [*p* = 0.009], and 0.64 [*p* = 0.00], respectively). At the 3-month follow-up, the IFCIG exhibited a significant reduction in the double support phase (ES: 0.68 [*p* = 0.003]). At the 2-month follow-up, the IFCIG showed significantly lower anteroposterior displacement and displacement area compared to the FIG (ES: 0.52 [*p* = 0.025] and 0.53 [*p* = 0.012], respectively), as seen in Table 1.

Regarding the participants’ scores on the Taiwanese version of the BPI (Table 1), at the 2-month follow-up, the IFCIG had significantly lower scores on the current pain item than did the FIG (ES: 0.62 [*p* = 0.023]). At the 3-month follow-up, the IFCIG had significantly lower scores on the least pain in the previous week, effect on normal work, and effect on interactions with other items than did the FIG (ES: 0.6 [*p* = 0.045], 0.61 [*p* = 0.017], and 0.6 [*p* = 0.029], respectively). Furthermore, in the IFCIG, scores on the average pain in the previous week, current pain, effect on normal work, and effect on interactions with others items were significantly lower at the 3-month follow-up than at baseline (ES: 0.61 [*p* = 0.004], 0.59 [*p* = 0.036], 0.55 [*p* = 0.012], and 0.51 [*p* = 0.028], respectively). Additionally, in the IFCIG, scores on the current pain and effect on normal work items were significantly lower at the 1-week and 2-month follow-ups than at baseline (ES: 0.61 [*p* = 0.021], 0.64 [*p* = 0.009], 0.51 [*p* = 0.041], and 0.58 [*p* = 0.008], respectively).

Regarding plantar pressure (Table 2), at baseline, peak pressure in the second metatarsal and medial heel was significantly lower when the IFCIG members wore IFCIs than when they wore FIs (ES: 0.51 [*p* = 0.032], 0.61 [*p* = 0.019], and 0.49 [*p* = 0.041], respectively). At baseline and the 1-week and 2-month follow-ups, peak pressure in the medial heel was significantly lower in the IFCIG than in the FIG (ES: 0.55 [*p* = 0.016], 0.49 [*p* = 0.031], and 0.5 [*p* = 0.048], respectively). At baseline, peak pressure in the lateral heel was significantly lower in the IFCIG than in the FIG (ES: 0.49 [*p* = 0.033]).

Regarding the contact area (Table 3), at baseline, the contact areas in the first metatarsal, second metatarsal, and third metatarsal were significantly smaller when the IFCIG members wore FIs than when they wore IFCIs (ES: 0.62 [*p* = 0.013], 0.49 [*p* = 0.044], 0.53 [*p* = 0.025], and 0.64 [*p* = 0.007], respectively). By contrast, the contact areas in the medial heel, lateral heel, and midfoot were significantly larger when the IFCIG members wore FIs than when they wore IFCIs (ES: 0.61 [*p* = 0.030], 0.64 [*p* = 0.012], 0.52 [*p* = 0.022], and 0.49 [*p* = 0.039], respectively). Furthermore, the contact areas in the medial heel and midfoot were significantly larger in the IFCIG than in the FIG (ES: 0.79 [*p* = 0.001], 0.57 [*p* = 0.013], and 0.55 [*p* = 0.019], respectively).

At the 1-week follow-up, the contact area in the fourth metatarsal was significantly smaller in the IFCIG than in the FIG (ES: 0.66 [*p* = 0.020] and 0.51 [*p* = 0.041]). By contrast, the contact areas in the medial heel and lateral heel were significantly larger in the IFCIG than in the FIG (ES: 0.52 [*p* = 0.035], 0.67 [*p* = 0.014], and 0.44 [*p* = 0.048], respectively).

At the 2-month follow-up, the contact area in the first metatarsal was significantly smaller in the IFCIG than in the FIG (ES: 0.49 [*p* = 0.043]). By contrast, the contact areas in the medial heel and midfoot were significantly larger in the IFCIG than in the FIG (ES: 0.6 [*p* = 0.012], 0.5 [*p* = 0.046], 0.53 [*p* = 0.030], and 0.56 [*p* = 0.048], respectively).

At the 3-month follow-up, the contact areas in the fourth and fifth toes, first metatarsal, and third metatarsal were significantly smaller in the IFCIG than in the FIG (ES: 0.55 [*p* = 0.036], 0.56 [*p* = 0.028], 0.61 [*p* = 0.037], and 0.64 [*p* = 0.017], respectively). However, these areas were significantly larger at the 2-month follow-up than at the 1-week follow-up (ES: 0.58 [*p* = 0.006] and 0.56 [*p* = 0.013], respectively).

## 4. Discussion

This study addresses the issue of plantar pain and altered gait characteristics in individuals with hallux valgus, a common deformity that can significantly impact daily activities. Given the limitations of commercially available insoles, which often lack sufficient customization to accommodate specific foot pathologies, this research aimed to evaluate the effectiveness of customized full-contact insoles made from polyester fabric sheets in improving plantar pressure distribution and gait dynamics.

In this study, the IFCIG showed significant reductions in anteroposterior displacement and displacement area at the 2-month follow-up. Gait analysis revealed shortened support and stance phases, along with an extended swing phase with prolonged IFCI use. Lower peak pressure was observed in specific regions such as the second metatarsal and medial heel in the IFCIG compared to the FIG. Additionally, pressure integrals for multiple regions were significantly lower in the IFCIG. Immediate IFCI use increased contact areas in the medial heel and midfoot, with prolonged use leading to increased forefoot pressure in the fourth and fifth metatarsals. The IFCIG also demonstrated significant reductions in BPI scores for least pain in the previous week and impact on daily activities. These findings underscore the positive effects of IFCIs on foot pressure distribution, gait characteristics, and pain perception, endorsing the manufacturing process used in this study.

After 2 months of IFCI wear, participants showed decreased static balance sway in the anteroposterior direction. This aligns with Fleischer et al.’s finding [23], indicating reduced center of gravity sway post-surgery in older hallux valgus patients, lowering fall risk [24]. Our >50-year-old participants exhibited improved balance, reducing sway during static standing after intervention, which is beneficial for hallux valgus individuals in fall prevention. Forefoot pain sufferers often shift weight to relieve discomfort, potentially causing foot pressure displacement. The observed reduction in displacement may result from IFCI’s pain relief.

Gait analysis showed that prolonged IFCI use decreased double support and stance phase durations while increasing the swing phase duration. Typically, the double support phase is shorter in the general population than in hallux valgus individuals [25], and corrective surgery for hallux valgus may further shorten it [23]. Additionally, individuals at higher fall risk tend to have shorter double support phases [26]. Our study indicates that wearing IFCIs can shorten the double support phase in middle-aged and older hallux valgus individuals, improving gait balance and reducing fall risk.

In this study, IFCIs significantly reduced peak pressure in the second metatarsal and medial heel. These findings support previous research, highlighting the benefits of customized insoles, including reducing soft tissue strain and foot pressure, enhancing support, and maintaining a correct foot position through arch support [27]. Studies indicate that customized full-contact insoles notably reduce peak foot pressure across the foot, including the heel, midfoot, metatarsal, and metatarsal head, along with reductions in peak pressure and pressure–time integrals [7]. In the present study, IFCIs significantly reduced peak pressure, particularly in the second metatarsal and heel during walking. This improvement ensures even pressure distribution, enhancing comfort and reducing pain and discomfort during walking in hallux valgus individuals [3,28].

IFCIs immediately increased contact areas in the medial heel and midfoot, consistent with prior studies [3,8,9]. Additionally, contact areas were larger in the IFCIG compared to the FIG. Arch-support insoles have been shown to notably increase medial midfoot contact area and foot pressure and slightly enhance medial forefoot and toe strength [7,8]. These insoles increased the medial midfoot contact area after 4 weeks, continuing to increase until week 8. Furthermore, contact areas in the second to fifth toes, metatarsals, and medial and lateral heels showed an increasing trend with arch-support insole use [9]. Such insoles promote even foot pressure distribution, reducing the soft-tissue damage risk during walking and potentially alleviating pain with prolonged use [29,30]. Customized full-contact insoles significantly increase contact areas in the bilateral heel, medial arch, and second to fifth toes, ensuring extensive and even foot pressure distribution [7].

With increased wear time, participants showed improvements in BPI scores, particularly in the least pain in the previous week and effect on daily activities items. Importantly, the IFCIG exhibited lower BPI scores than the FIG, highlighting the effectiveness of IFCIs in pain relief. This aligns with Coheña-Jiménez et al.’s findings [31] demonstrating that arch-support insoles reduce foot pain and enhance foot function with lasting effects. However, discontinuing their use may lead to recurrence of symptoms. Arch-support insole usage notably improved individuals’ pain scores, surpassing extracorporeal shockwave therapy in pain reduction, foot function enhancement, and foot health maintenance [32]. Reduced pain improves quality of life, enhancing physical activity like walking speed and stair-climbing, with insoles aiding daily activities by relieving pain, boosting mobility, and reducing injury risks [27,33].

Previous studies have shown that participants may wear orthotics for weeks or even years [34,35], yet there is limited literature addressing whether the effectiveness of these orthotics diminishes over time due to prolonged use [36]. The manufacturing process and materials used for the full-contact orthotics in this study allow for rapid production of new orthotics in a short period. This capability effectively eliminates concerns regarding the potential decline in orthotic efficacy due to wear over time.

This study has limitations. We only included individuals with self-reported forefoot pain and hallux valgus, potentially limiting the generalizability of our findings. Participant arch types were not specified, possibly impacting result interpretation. Analytical models were not adjusted for participants’ daily footwear choices, potentially introducing confounding effects. The mean participant age was 54 years, and no sex-specific analysis was conducted, indicating potential variations between age groups and sexes.

## 5. Conclusions

Customized full-contact insoles, crafted from a combination of soft, perforated ethylene vinyl acetate material and polyester fabric sheets of thermoplastic extruded materials, demonstrated notable enhancements in center of pressure deviation, gait characteristics, and foot pressure distribution among individuals suffering from forefoot pain associated with hallux valgus. These findings underscore the potential of custom insoles in relieving foot discomfort in this demographic and underscore the advantages of tailored support. The study results suggests that customized full-contact insoles hold promise in addressing foot issues related to hallux valgus. To fully elucidate the efficacy of custom insoles in alleviating foot pain and enhancing quality of life among individuals with hallux valgus, future research should expand sample sizes, encompass diverse populations, and account for various arch types.

## Figures and Tables

**Table 1:** Gait, balance parameters and brief pain inventory scores.

Time Points:	0 Week			After 1 Week		After 2 Months		After 3 Months	
Groups:	Control Group (FIG)	Experimental Group (IFCIG)	Experimental Group (IFCIG)	Control Group (FIG)	Experimental Group (IFCIG)	Control Group (FIG)	Experimental Group (IFCIG)	Control Group (FIG)	Experimental Group (IFCIG)
Insoles:	FI	FI	IFCI	FI	IFCI	FI	IFCI	FI	IFCI
Gait									
Velocity (cm/s)	111.63 (18.89)	112.39 (16.67)	111.53 (15.66)	112.38 (19.12)	113.27 (15.67)	116.21 (17.7)	115.03 (15.12)	113.85 (17.09)	116.23 (14.75)
Cadence (step/min)	109.18 (8.43)	111.21 (8.38)	111.04 (8.09)	109.79 (8.49)	112.58 (8.21)	110.52 (8.64)	113.11 (8.02)	110.03 (7.87)	113.81 (8.38)
Swing% of Cycle (%)	35.74 (1.83)	35.99 (1.80)	35.78 (1.66) ^a,e^	35.58 (1.79)	36.27 (1.64) ^a^	36.01 (1.79)	36.46 (1.74) ^e^	36.13 (1.59)	36.22 (1.50)
Stance % of Cycle (%)	64.26 (1.83)	64.01 (1.80)	64.22 (1.66) ^a,e^	64.43 (1.79)	63.73 (1.64) ^a^	63.99 (1.79)	63.54 (1.74) ^e^	63.88 (1.59)	63.78 (1.50)
Single Support% Cycle (%)	36.36 (1.08)	35.52 (2.08)	35.77 (1.85)	35.91 (1.21)	35.99 (1.91)	36.44 (0.99)	36.40 (1.98)	36.22 (1.27)	36.48 (2.15)
Double Support% Cycle (%)	27.93 (2.52)	28.46 (3.83)	28.47 (3.38) ^d,e^	28.41 (2.56)	27.89 (3.18)	27.50 (2.42)	27.29 (3.40) ^e^	27.61 (2.40)	27.62 (3.52) ^d^
Balance									
Medial-lateral Displacement (cm)	0.72 (0.46)	0.63 (0.33)	0.57 (0.27)	0.78 (0.41)	0.70 (0.29)	1.00 (0.87)	0.64 (0.30)	0.75 (0.54)	0.67 (0.32)
Anterior-posterior Displacement (cm)	2.93 (1.11)	2.81 (0.83)	2.67 (0.78)	3.01 (0.86)	2.87 (0.83)	3.40 (1.38) *	2.50 (0.94) *	2.86 (0.90)	2.68 (0.76)
Displacement Area (cm^2^)	2.01 (2.60)	1.54 (1.07)	1.30 (0.85)	2.18 (1.89)	1.74 (1.08)	2.17 (1.38) *	1.38 (1.15) *	1.94 (1.94)	1.65 (1.30)
Pain severity									
Most severe	4.12 (2.32)		4.41 (2.39)	3.65 (2.91)	3.59 (2.79)	4.41 (3.03)	3.91 (2.91)	4.06 (2.31)	2.5 (2.68)
Least	1.06 (1.3)		0.64 (0.93)	0.88 (1.18)	0.77 (0.95)	1 (1.28)	0.77 (1.13)	1.41 (1.37) *	0.36 (0.77) *
Average	2.41 (1.78)		3.14 (2.14) ^d^	2.15 (1.89)	2.14 (1.96)	2.59 (1.97)	2.05 (1.74)	2.41 (1.88)	1.68 (2.1) ^d^
Now	1.41 (1.65)		1.68 (1.74) ^a,d,e^	1.47 (1.72)	0.95 (1.33) ^a^	1.53 (1.79) *	0.59 (0.94) *^,e^	1.18 (1.72)	0.55 (1.2) ^d^
Pain interference									
General activity	0.41 (1.24)		1.27 (1.84)	0.82 (1.58)	0.86 (1.58)	0.94 (1.7)	0.91 (1.65)	0.59 (1.37)	0.45 (0.89)
Mood	1.88 (2.08)		2.45 (2.02)	1.41 (2.22)	1.77 (2.11)	1.94 (2.18)	1.32 (1.77)	2.12 (2.4) *	0.95 (1.87) *
Walking ability	2.88 (2.59)		2.82 (2.9)	2.24 (2.46)	1.91 (2.17)	2.56 (3.07)	2.27 (2.43)	2.47 (2.5)	1.55 (1.92)
Normal work	2.53 (2.33)		2.64 (2.69) ^a,d,e^	1.94 (2.36)	1.36 (2.33) ^a^	2.53 (2.5)	1.5 (2.13) ^e^	2.41 (2.7) *	0.59 (1.78) * ^d^
Relationship	0.88 (2.27)		0.68 (1.26) ^d^	1 (1.88)	0.55 (1.3)	0.71 (1.67)	0.41 (0.89)	0.76 (1.96) *	0 (0) *^, d^
Sleep	1.18 (1.98)		0.77 (1.31)	1.35 (2.4)	0.5 (1.03)	2.06 (2.58)	0.68 (1.77)	1.18 (2.04)	0.5 (1.7)
Enjoyment of life	1.47 (2.35)		2.18 (2.27)	1.47 (2.12)	1.5 (1.78)	2.24 (2.62)	1.73 (2.56)	1.53 (2.28)	1.18 (2.04)

a: Significance differences between 0 weeks and 1 week; d: Significance differences between 0 weeks and 3 months; e: Significance differences between 0 weeks and 2 months; FI: Flat insoles; IFCI: Innovative full-contact insoles; *: Significance differences between the experimental and control group. *p* < 0.05.

**Table 2:** Peak pressure (Kpa).

Time Points:	0 Week			After 1 Week		After 2 Months		After 3 Months	
Groups:	Control Group (FIG)	Experimental Group (IFCIG)	Experimental Group (IFCIG)	Control Group (FIG)	Experimental Group (IFCIG)	Control Group (FIG)	Experimental Group (IFCIG)	Control Group (FIG)	Experimental Group (IFCIG)
Insoles:	FI	FI	IFCI	FI	IFCI	FI	IFCI	FI	IFCI
T1	402.04 (199.50)	329.30 (235.15)	310.07 (251.97)	408.29 (253.85)	419.86 (298.31)	420.80 (296.93)	324.01 (176.14)	399.89 (362.81)	352.92 (188.07)
T2	241.68 (173.25)	153.29 (56.99)	174.00 (96.15)	419.05 (634.73)	199.40 (122.24)	214.06 (183.58)	141.24 (64.28)	168.60 (107.67)	201.97 (122.33)
T3	185.33 (142.32)	144.14 (69.14)	145.33 (43.08)	145.58 (54.37)	174.61 (77.00)	178.15 (108.01)	130.58 (43.22)	174.25 (112.80)	165.72 (71.68)
T45	145.31 (92.57)	116.45 (50.66)	125.66 (79.23)	133.73 (73.49)	128.64 (40.73)	157.86 (126.75)	120.79 (44.34)	140.19 (119.18)	127.15 (89.03)
M1	325.26 (215.06)	290.95 (175.61)	283.63 (188.18)	304.51 (137.32)	386.12 (304.80)	328.64 (211.92)	289.64 (151.68)	288.45 (181.93)	336.54 (223.12)
M2	320.93 (131.77)	319.00 (139.02) ^+^	279.55 (128.59) ^+^	446.34 (319.67)	321.19 (183.96)	382.24 (167.90)	314.64 (172.05)	349.11 (165.43)	320.43 (143.76)
M3	393.00 (169.32)	350.41 (116.99)	353.01 (123.00)	480.38 (273.71)	390.51 (202.94)	421.30 (176.20)	376.32 (151.86)	416.21 (209.25)	431.03 (198.83)
M4	284.86 (173.37)	211.76 (92.45)	224.39 (88.29)	264.03 (141.50)	240.07 (142.46)	255.75 (95.10)	243.68 (80.54)	255.20 (145.39)	289.86 (154.25)
M5	242.35 (263.01)	147.41 (73.82)	150.43 (77.47)	146.93 (60.99)	162.94 (109.82)	171.75 (72.95)	179.34 (98.58)	154.45 (79.32)	197.67 (128.33)
MH	351.91 (115.52) *	366.02 (150.85) ^+^	261.95 (138.51) ^+,^*	362.34 (137.07) *	295.24 (194.16) *	372.31 (132.45) *	262.11 (93.74) *	332.10 (148.00)	263.60 (100.22)
LH	311.56 (98.71) *	304.50 (87.67)	237.00 (95.03) *	302.78 (115.29)	295.87 (178.27)	324.1 (113.19)	251.16 (74.07)	287.73 (119.56)	257.71 (78.91)
MF	134.49 (50.11)	157.74 (99.49)	169.59 (103.97)	192.01 (185.55)	210.01 (223.26)	122.58 (47.96)	222.91 (200.17)	135.11 (96.06)	209.01 (224.07)

FI: Flat insoles; IFCI: Innovative full-contact insoles; T1: The hallux; T2–5: Toes 2–5; M1–5: Metatarsals 1–5; MH: Medial heel; LH: Lateral heel; MF: Midfoot; *: Significance differences between the experimental and control group. ^+^: Significance differences between the experimental group wearing IFCIs and FIs. *p* < 0.05.

**Table 3:** Contact Area (cm^2^).

Time Points:	0 Week			After 1 Week		After 2 Months		After 3 Months	
Groups:	Control Group (FIG)	Experimental Group (IFCIG)	Experimental Group (IFCIG)	Control Group (FIG)	Experimental Group (IFCIG)	Control Group (FIG)	Experimental Group (IFCIG)	Control Group (FIG)	Experimental Group (IFCIG)
Insoles:	FI	FI	IFCI	FI	IFCI	FI	IFCI	FI	IFCI
T1	4.74 (1.86)	5.07 (2.15)	4.77 (2.33)	4.85 (1.76)	4.55 (1.93)	5.04 (1.81)	4.56 (1.98)	5.08 (1.97)	4.29 (1.83)
T2	2.97 (0.87)	2.95 (1.07)	3.15 (0.94)	2.68 (0.88)	2.66 (1.07)	2.8 (0.87)	2.82 (1.03)	2.8 (0.54)	2.90 (1.10)
T3	2.67 (0.83)	2.99 (0.94)	2.79 (1.12)	2.51 (0.94)	2.26 (0.68)	2.59 (0.91)	2.31 (1.04)	2.83 (0.89)	2.27 (1.00)
T45	1.88 (0.77)	2.25 (0.98)	2.24 (1.27)	1.66 (0.66)	1.59 (0.71)	1.97 (0.85)	1.75 (1.08)	1.91 (0.71) *	1.39 (0.86) *
M1	9.17 (2.09)	9.04 (2.38) ^+^	7.90 (2.47) ^+^	9.48 (1.87)	7.95 (2.31)	9.77 (2.12) *	8.06 (2.32) *	9.92 (2.53) *	7.38 (2.35) *
M2	5.12 (1.60)	5.63 (1.30) ^+^	5.19 (1.01) ^+^	5.46 (1.25)	4.95 (1.00)	5.68 (0.88)	5.22 (1.02)	5.68 (1.19)	4.94 (1.40)
M3	6.16 (1.20)	6.44 (1.44) ^+^	5.92 (1.11) ^+^	6.36 (1.06)	5.64 (1.05)	6.58 (0.94)	6.08 (1.01)	6.69 (1.05) *	5.84 (1.37) *
M4	6.36 (1.17)	6.16 (1.66)	5.80 (1.18)	6.35 (1.58) *	5.34 (1.19) *	6.62 (1.3)	6.07 (1.33)	6.53 (1.23)	5.78 (1.47)
M5	6.68 (2.05)	6.18 (2.08)	5.98 (1.57)	6.36 (2.53)	4.81 (1.83)	6.86 (2.06)	6.25 (1.73)	6.74 (1.41)	5.81 (2.05)
MH	13.99 (1.54) *	15.00 (3.25) ^+^	17.91 (3.88) ^+,^*	14.42 (1.73) *	16.46 (2.25) *	14.47 (2.00) *	17.27 (2.52) *	14.82 (2.70)	16.89 (3.26)
LH	14.56 (1.21)	15.94 (3.70) ^+^	18.71 (3.94) ^+^	14.45 (1.98) *	16.93 (3.34) *	15.12 (1.95)	17.25 (3.60)	15.10 (2.71)	17.79 (4.35)
MF	13.51 (6.93) ^a,^*	17.09 (10.20) ^+^	23.26 (12.43 ) ^a,+,^*	14.55 (8.26) ^a,b^	14.20 (6.86) ^a,b^	13.88 (9.83) ^b^	17.79 (9.66) ^b^	16.58 (10.12)	16.01 (9.87)

FI: Flat insoles; IFCI: Innovative full-contact insoles; T1: The hallux; T2–5: Toes 2–5; M1–5: Metatarsals 1–5; MH: Medial heel; LH: Lateral heel; MF: Midfoot; a: Significance differences between 0 weeks and 1 week; b: Significance differences between 1 week and 2 months; *: Significance differences between the experimental and control group. ^+^: Significance differences between the experimental group wearing IFCIs and FIs. *p* < 0.05.

## Data Availability

Dataset available on request from the authors.

## Glossary

IFCIs	customized innovative full-contact insoles
FIs	flat insoles
IFCIG	experimental group, IFCI group
FIG	control group, FI group
BPI	Brief Pain Inventory
T1	hallux
T2	second toe
T3	third toe
T45	fourth and fifth toes
M1	first metatarsal
M2	second metatarsal
M3	third metatarsal
M4	fourth metatarsal
M5	fifth metatarsal
MH	medial heel
LH	lateral heel
MF	midfoot
